# Clinical and Laboratory Factors Associated with Exchange Transfusion in Neonates with Severe Hyperbilirubinaemia: A Retrospective Cohort Study

**DOI:** 10.3390/jcm15218762

**Published:** 2026-11-09

**Authors:** Aslıhan İzol, Selçuk Yazıcı, Sümeyya Ercan

**Affiliations:** 1 Department of Paediatrics, Şanlıurfa Education and Research Hospital, 63200 Şanlıurfa, Türkiye; aslihan_zengin@hotmail.com (A.İ.); drsumeyyaercan@gmail.com (S.E.); 2 Department of Paediatrics, Private Hospital, Osmangazi Cad. No:34 Sarigazi, Sancaktepe, 34885 İstanbul, Türkiye

**Keywords:** neonatal hyperbilirubinaemia, exchange transfusion, total serum bilirubin, retrospective cohort, treatment-indication bias, model validation

## Abstract

**Background and Objectives:** Exchange transfusion (ET) remains a critical intervention for severe neonatal hyperbilirubinaemia, yet laboratory associations with ET receipt require careful interpretation. We examined characteristics associated with ET and post-procedure laboratory changes. **Materials and Methods:** This retrospective tertiary-centre cohort included 98 neonates evaluated between January 2020 and December 2025 (65 received ET; 33 did not). A four-covariate logistic regression model assessed associations with ET receipt. Discrimination, bootstrap optimism, cross-validated calibration and an exploratory creatinine-containing model were examined. **Results:** Peak total serum bilirubin (TSB) was higher among ET recipients than non-recipients (33.69 ± 6.31 versus 26.19 ± 3.64 mg/dL; *p* < 0.001). In the primary model (*n* = 98), peak TSB was associated with ET receipt (adjusted odds ratio 1.31 per mg/dL, 95% confidence interval 1.16–1.48; *p* < 0.001). The apparent area under the receiver operating characteristic curve (AUC) was 0.860, decreasing to 0.837 after bootstrap optimism correction. An exploratory six-covariate model (*n* = 97) yielded an apparent AUC of 0.874 and an optimism-corrected AUC of 0.836. Creatinine showed an adjusted association (odds ratio 1.58 per 0.1 mg/dL, 95% confidence interval 1.07–2.32), but its incremental AUC was uncertain (difference 0.014, 95% confidence interval −0.022 to 0.053). Following ET, bilirubin, platelet count and total calcium decreased. **Conclusions:** Peak TSB strongly distinguished infants who received ET, but this association partly reflects the bilirubin-based indication for treatment. Creatinine did not demonstrate a reliable incremental discrimination benefit. These findings reinforce guideline-based ET assessment and post-exchange monitoring, not the adoption of new treatment thresholds or prediction tools.

## 1. Introduction

Neonatal unconjugated hyperbilirubinaemia is one of the most frequently encountered conditions during the early postnatal period, affecting approximately 60% of term and 80% of preterm infants [1]. Although most cases follow a benign and self-limiting course, severe hyperbilirubinaemia remains an important cause of preventable neonatal morbidity and mortality worldwide. Excessive bilirubin exposure may result in acute bilirubin encephalopathy and, in its most severe form, kernicterus spectrum disorder, both of which are associated with irreversible neurological impairment and lifelong disability [1,2,3,4].

The introduction of evidence-based phototherapy protocols has substantially reduced the incidence of bilirubin-induced neurological injury and the need for exchange transfusion (ET) [2,4]. Nevertheless, ET remains a critical intervention for infants with bilirubin concentrations meeting gestational-age-, postnatal-age- and neurotoxicity-risk-adjusted treatment thresholds; inadequate response to intensive phototherapy; or clinical evidence of acute bilirubin encephalopathy [5,6,7]. Although potentially life-saving, ET is an invasive procedure associated with electrolyte disturbances, thrombocytopenia, haemodynamic instability, vascular-access complications and infection [5]. Consequently, understanding the clinical context in which ET is undertaken and anticipating post-procedural complications remain important priorities.

From a transfusion-medicine perspective, ET requires carefully selected, compatible blood components and is commonly performed as a double-volume exchange through suitable vascular access, with cardiorespiratory, haematological and metabolic monitoring. The Turkish Neonatal Society guidance addresses blood-product selection, procedural technique and surveillance for complications [7,8]. In our centre, blood-component selection, double-volume exchange, vascular access, and calcium surveillance and supplementation when clinically indicated formed part of standard institutional practice. These procedural safeguards are relevant because ET may be followed by changes in bilirubin, platelet count and calcium concentrations [5,9,10,11].

Previous investigations have primarily focused on the epidemiology, aetiology and outcomes of severe neonatal hyperbilirubinaemia, whereas relatively few studies have comprehensively evaluated demographic characteristics, haemolytic markers and routinely available laboratory parameters together [9,12,13,14]. Furthermore, contemporary evidence describing laboratory alterations following ET remains limited despite their direct relevance to post-procedural monitoring and supportive care [5,9,10,11]. The interpretation of associations with ET requires particular caution because bilirubin concentration is itself central to the treatment decision, alongside gestational age, postnatal age and neurotoxicity risk factors [2,7].

The present study aimed to compare the demographic, clinical and laboratory characteristics of neonates with clinically significant hyperbilirubinaemia according to whether ET was performed; examine adjusted associations with receipt of ET and the stability of exploratory discrimination models; and characterise paired laboratory changes following ET. These analyses seek to inform the interpretation of routine clinical measurements and post-exchange surveillance, without proposing an alternative to guideline-based treatment thresholds.

## 2. Materials and Methods

### 2.1. Study Design and Setting

This retrospective observational cohort study was conducted in the Neonatal Intensive Care Unit of Şanlıurfa Training and Research Hospital, a tertiary referral centre in southeastern Türkiye, between 1 January 2020 and 31 December 2025. Medical records of neonates admitted with severe unconjugated hyperbilirubinaemia were retrospectively reviewed. The study was designed and reported in accordance with the Strengthening the Reporting of Observational Studies in Epidemiology (STROBE) statement.

### 2.2. Ethical Approval

The study was approved by the Harran University Non-Interventional Clinical Research Ethics Committee (Approval date: 16 June 2026, Approval No. HÜ-GOEK-2026-00088). As the study used anonymised routinely collected data, the requirement for written informed consent was waived. The study complied with the Declaration of Helsinki and its subsequent amendments.

### 2.3. Study Population and Eligibility

The analytical cohort comprised neonates managed at the tertiary NICU for clinically significant unconjugated hyperbilirubinaemia between 2020 and 2025. Eligibility was determined by clinical severity and need for specialist evaluation, not by a universal peak TSB > 25 mg/dL criterion. Of 107 records screened, nine were excluded: two because of conjugated hyperbilirubinaemia, two because of suspected major congenital or chromosomal disorders, one because of suspected metabolic disease, and four because they were duplicate records. The remaining 98 infants were included in the analytical cohort, including 18 with recorded peak TSB ≤ 25 mg/dL.

No formal a priori sample-size calculation was performed. All 98 eligible records remaining after screening were analysed. Because this was a tertiary referral cohort, the proportion receiving ET must not be interpreted as a population treatment rate.

### 2.4. Clinical Management and Data Collection

Clinical management was based on the Turkish Neonatal Society guidance applicable during the study period, including the 2023 update in later years [7] and earlier guidance in preceding years [8]. ET decisions considered gestational and postnatal age, TSB concentration and trajectory, neurotoxicity risk factors, neurological findings and response to intensive phototherapy. Institutional standard practice involved compatible blood-component selection, double-volume exchange, appropriate vascular access, and calcium monitoring and supplementation when clinically indicated. Routine prophylactic calcium administration should not be inferred from this description. Exact exchange duration, actual volumes, product details and individual protocol deviations were not consistently recoverable.

Data were extracted from electronic medical, laboratory and treatment records using a standardised form. Variables included gestational age, birth weight, postnatal age, ABO and Rh incompatibility, direct Coombs positivity, IVIG administration, bilirubin encephalopathy and mortality. Baseline laboratory variables comprised peak TSB, haemoglobin, platelet count, blood urea, serum creatinine, albumin, sodium, calcium and C-reactive protein (CRP). Corresponding post-ET measurements were collected for ET-treated infants. Laboratory measurements were obtained from the hospital’s routine clinical laboratory records, and the measurements used for analysis corresponded to the closest available values before ET and the first available measurements following ET. Serum creatinine was measured by the Jaffé method using the CREJ2 reagent assay on a cobas c analyser (Roche Diagnostics). Details of any sample-specific procedures for identifying or correcting icteric interference could not be verified retrospectively. Exact sampling times relative to admission, intravenous fluids and exchange transfusion were not consistently recoverable, and the interval from completion of ET to the first post-ET sample could not be reliably calculated.

### 2.5. Definitions and Outcomes

ABO and Rh incompatibility were classified from documented maternal–infant blood-group records; direct Coombs positivity was analysed separately as evidence of antibody-mediated red-cell sensitisation rather than assumed proof of clinically significant haemolysis. A standardised haemolysis severity score, reticulocyte trajectory and G6PD status were not consistently available. Acute bilirubin encephalopathy was based on the treating clinician’s recorded neurological assessment; standardised BIND scoring and reliable timing relative to ET could not be reconstructed. Peak TSB was the highest recorded concentration before ET in treated infants and before improvement in non-ET infants, producing different outcome-dependent observation windows.

The primary outcome was documented receipt of ET during admission. Secondary descriptive outcomes were recorded encephalopathy, mortality, between-group differences and paired laboratory changes. Regression and ROC analyses were exploratory analyses of treatment received, not prospective clinical prediction.

Potential sources of bias included retrospective case ascertainment, referral and selection effects and incorporation of bilirubin concentration into ET decisions. The study screened 107 records, excluded nine for prespecified clinical or record-integrity reasons and analysed 98 eligible infants. The retrospective design does not establish whether all potentially eligible neonates in the catchment population were referred to this centre.

### 2.6. Statistical Analysis

Statistical analyses were conducted using IBM SPSS Statistics (version 23) and Python 3.11 (statsmodels and scikit-learn). Continuous variables were summarised using mean ± SD or median (IQR) according to distribution; Welch’s *t*-test was used for approximately symmetric variables when variances could differ, and Mann–Whitney U tests for skewed variables. Categorical variables were compared using χ^2^ or Fisher’s exact tests as appropriate. Group comparisons were exploratory and not adjusted for multiplicity.

The primary adjusted association analysis used a clinically interpretable four-covariate logistic model (peak TSB, postnatal age, ABO incompatibility and direct Coombs positivity; *n* = 98), excluding urea and creatinine to limit model complexity and prioritise clinically interpretable covariates. A six-covariate model, additionally including urea and Jaffé creatinine, was fitted as an exploratory sensitivity analysis (*n* = 97); an otherwise identical five-covariate model excluding creatinine was fitted to the same 97 infants. Model specifications were determined during analysis and were not prospectively prespecified. Odds ratios, 95% confidence intervals, likelihood-ratio statistics and McFadden’s pseudo-R^2^ were calculated. Predictor VIFs were assessed on the design matrix. Exploratory quadratic-term likelihood-ratio tests assessed departures from linearity in the logit, without claiming that the assumption was universally met.

ROC AUCs were calculated within the development cohort. To quantify optimism, 350 bootstrap samples were used for the four- and six-covariate models, refitting each model and subtracting mean bootstrap optimism from apparent AUC. Five-fold stratified out-of-fold predictions were used for additional discrimination and calibration assessment. The incremental AUC of creatinine was assessed using paired, stratified bootstrap resampling (3000 replicates) of predictions from otherwise identical five- and six-covariate models in the same 97 infants; this describes uncertainty in the observed AUC difference, not external validation. The 31.53 mg/dL Youden cut-off and 27.02 mg/dL descriptive comparator were evaluated as cohort-specific illustrations, not treatment thresholds. Regression used complete cases, with missingness reported by covariate. Paired pre/post ET laboratory tests followed the original analysis; exact sampling intervals were unavailable. All tests were exploratory and two-sided.

## 3. Results

### 3.1. Study Population

Of 107 screened records, nine were excluded (two conjugated hyperbilirubinaemia, two suspected major congenital/chromosomal disorders, one suspected metabolic disease and four duplicates). The final cohort included 98 infants: 65 (66.3%) received ET and 33 (33.7%) did not. Peak TSB ranged from 20.47 to 48.60 mg/dL in the ET group and from 18.90 to 33.00 mg/dL in the non-ET group. Six ET infants and 12 non-ET infants had recorded peak TSB ≤ 25 mg/dL. The study-flow summary reports all screened and excluded records and the final treatment groups.

Study flow: Records screened (*n* = 107) → Excluded (*n* = 9): conjugated hyperbilirubinaemia (*n* = 2); suspected major congenital/chromosomal disorder (*n* = 2); suspected metabolic disease (*n* = 1); duplicate records (*n* = 4) → Included in analysis (*n* = 98) → ET (*n* = 65); no ET (*n* = 33).

### 3.2. Baseline Demographic Characteristics

Baseline demographic and clinical characteristics are summarised in Table 1. Gestational age, birth weight and postnatal age at admission did not differ significantly between groups when analysed using distribution-appropriate non-parametric tests. Peak total serum bilirubin was substantially higher in infants undergoing ET.

Mean peak total bilirubin was 33.69 ± 6.31 mg/dL in the ET group versus 26.19 ± 3.64 mg/dL in the non-ET group (*p* < 0.001).

## 4. Aetiological and Clinical Characteristics

The distribution of major aetiological and clinical characteristics according to ET status is presented in Table 2. ABO incompatibility and direct Coombs positivity were numerically more frequent in the non-ET group, but neither difference reached statistical significance. Rh incompatibility and IVIG administration were also not significantly associated with ET. Bilirubin encephalopathy was significantly more frequent among infants undergoing ET, whereas in-hospital mortality did not differ significantly between groups.

ABO incompatibility was observed in 20 of 33 infants (60.6%) in the non-ET group and 27 of 65 infants (41.5%) in the ET group, demonstrating a non-significant trend towards lower frequency among infants requiring exchange transfusion (*p* = 0.089). Similarly, direct antiglobulin (Coombs) positivity was identified in 36.4% of infants managed without ET and 20.0% of those undergoing ET (*p* = 0.091). Rh incompatibility was not associated with ET requirement (12.1% vs. 15.4%, *p* = 0.767).

Bilirubin encephalopathy occurred in 2 of 33 non-ET infants (6.1%) and in 23 of 65 ET infants (35.4%; *p* = 0.001).

## 5. Laboratory Findings

Baseline laboratory findings are summarised in Table 3. Haemoglobin and calcium were approximately normally distributed and were compared using Welch’s independent-samples *t*-test; skewed laboratory variables were compared using the Mann–Whitney U test.

Blood urea concentration was significantly higher in the ET group (median 34.2 vs. 21.7 mg/dL, *p* = 0.009). C-reactive protein was also higher (median 1.40 vs. 0.89 mg/L, *p* = 0.039). No statistically significant between-group differences were observed for haemoglobin, platelet count, creatinine, albumin, sodium or calcium.

Missingness audit: All four primary-model covariates (postnatal age, peak TSB, ABO incompatibility and direct Coombs status) were recorded for 98/98 infants. Urea and creatinine were each missing for one infant, leaving 97/98 complete cases for the exploratory five- and six-covariate models. Complete-case analysis was used because only one record was affected; imputation was not undertaken. Other outcomes were analysed using their available observations.

## 6. Multivariable Associations with Receipt of Exchange Transfusion

The primary model included peak TSB, postnatal age, ABO incompatibility and direct Coombs positivity (*n* = 98). Peak TSB remained associated with receipt of ET (adjusted OR 1.31 per mg/dL, 95% CI 1.16–1.48; *p* < 0.001), whereas the other variables were not independently associated. The likelihood-ratio χ^2^ was 37.85 (*p* < 0.001), McFadden’s pseudo-R^2^ was 0.302 and the apparent AUC was 0.860. VIFs were 1.03–1.24, indicating that the originally reported VIF of 13.05 was not reproduced in the corrected model matrix.

In the exploratory six-covariate model (*n* = 97), peak TSB (adjusted OR 1.37, 95% CI 1.18–1.59) and creatinine (adjusted OR 1.58 per 0.1 mg/dL, 95% CI 1.07–2.32; *p* = 0.021) were associated with ET receipt. Its apparent AUC was 0.874 and VIFs were 1.03–1.98. The otherwise identical model without creatinine (*n* = 97) had an apparent AUC of 0.859. The paired AUC difference was 0.014 (bootstrap 95% CI −0.022 to 0.053), providing no clear evidence of an increment in discrimination. A nested likelihood-ratio test favoured including creatinine (χ^2^ = 6.36, *p* = 0.012), but this exploratory in-sample association does not establish incremental clinical utility.

Exploratory quadratic-term checks suggested departure from linearity for postnatal age in both models (primary-model *p* = 0.002) and for peak TSB in the primary model (*p* = 0.017). These tests were not multiplicity-adjusted and the cohort was too small for a stable flexible multivariable model. Consequently, linear odds ratios are descriptive approximations rather than proof of constant effects across the full observed range.

The primary-model coefficients are shown in Table 4, with the six-covariate sensitivity estimates presented separately in the same table. Treatment-indication bias, uncertain sampling times and possible nonlinear associations limit the interpretation of all coefficients.

### Model Performance and ROC Analysis

Internal-validation summary (five-fold out-of-fold): primary four-covariate model, AUC 0.831, calibration intercept 0.10 and slope 0.80; exploratory six-covariate model, AUC 0.822, calibration intercept 0.15 and slope 0.72. These are internal estimates and should not be interpreted as external validation.

The primary four-covariate model had an apparent AUC of 0.860 and bootstrap optimism-corrected AUC of 0.837 (350 successful bootstrap resamples). Five-fold out-of-fold AUC was 0.831; the corresponding calibration intercept was 0.10 and slope 0.80. For the exploratory six-covariate model, apparent AUC was 0.874, optimism-corrected AUC 0.836 and five-fold out-of-fold AUC 0.822, with calibration intercept 0.15 and slope 0.72. The lower out-of-fold calibration slopes suggest some overfitting. The same-sample five-versus-six-covariate paired AUC difference was 0.014 (95% bootstrap CI −0.022 to 0.053) (Figure 1). Figure 2 depicts TSB alone, not the multivariable model.

At the cohort-derived TSB threshold ≥31.53 mg/dL, sensitivity was 67.7% (44/65) and specificity 93.9% (31/33). At the illustrative comparator ≥27.02 mg/dL, sensitivity was 83.1% (54/65) and specificity 57.6% (19/33). The latter value was retained to illustrate the sensitivity–specificity trade-off, not because it is a universal guideline threshold. Neither threshold should determine ET independently of gestational age, postnatal age, neurotoxicity risk factors or clinical assessment.

## 7. Changes in Laboratory Parameters Following Exchange Transfusion

Paired analyses were performed among infants who underwent ET to evaluate changes in laboratory parameters before and after the procedure (Table 5). Complete paired data were available for 65 infants, except for platelet count, for which 64 paired observations were available.

A marked reduction in total serum bilirubin was observed following ET, from 33.69 to 14.69 mg/dL (mean paired change −18.99 mg/dL, 95% CI −20.39 to −17.59, *p* < 0.001).

Platelet counts also declined significantly, from 318.0 to 119.7 ×10^3^/µL (mean paired change −198.30 ×10^3^/µL, 95% CI −236.41 to −160.18, *p* < 0.001). Serum calcium decreased significantly on paired analysis (median 9.40 to 8.49 mg/dL; median paired change −0.82 mg/dL, *p* = 0.002).

No statistically significant changes were observed in haemoglobin (mean paired change −0.51 g/dL, 95% CI −1.34 to 0.32, *p* = 0.234) or sodium (median paired change −1.0 mmol/L, *p* = 0.187).

## 8. Discussion

The central finding is an association between peak TSB and receipt of ET, a clinical action determined in part by bilirubin concentration. A primary four-covariate model yielded apparent AUC 0.860, but optimism-corrected AUC was 0.837 and five-fold out-of-fold AUC was 0.831. The exploratory creatinine-containing model showed an adjusted association, but no clear incremental AUC improvement over an otherwise identical model. These findings should be interpreted as descriptive and hypothesis-generating.

ET decisions integrate bilirubin concentration with gestational age, postnatal age, neurotoxicity risk factors, neurological status and response to intensive phototherapy [2,7,15]. The strong TSB association is therefore susceptible to incorporation and confounding-by-indication bias. Furthermore, peak TSB was ascertained over different clinical observation windows according to ET status. The present models cannot support prospective prediction or replace treatment guidance.

The apparent TSB-only AUC was 0.845, and the exploratory ≥31.53 mg/dL cut-off had sensitivity 67.7% and specificity 93.9%. The additional ≥27.02 mg/dL comparator was evaluated solely to show the sensitivity–specificity trade-off (83.1% and 57.6%, respectively); it was not intended as a fixed clinical threshold. Treatment thresholds depend on age and neurotoxicity risk, and this cohort-derived cut-off must not be applied to patient care [2,7]. Published ET cohorts have reported mean peak bilirubin concentrations of approximately 25–28 mg/dL [5,6,9,16,17,18], while concentrations above 30 mg/dL have been associated with a greater burden of acute bilirubin encephalopathy and kernicterus spectrum disorders [9,14,16,19,20]. These studies provide clinical context for the high bilirubin concentrations observed here, but differences in referral pathways, eligibility criteria and treatment decisions preclude direct comparison of ROC-derived thresholds.

The adjusted creatinine association in the six-covariate model (OR 1.58 per 0.1 mg/dL, 95% CI 1.07–2.32) contrasts with the non-significant univariable comparison. Its interpretation is limited by exploratory selection, possible nonlinear relations, the small sample, uncertain sampling times and bilirubin interference with the Jaffé assay. Although the nested likelihood-ratio comparison was statistically significant, the paired AUC difference had a confidence interval spanning zero; neither analysis establishes clinically useful added discrimination. Previous reports have described renal complications after ET—including uncommon severe cases requiring dialysis [5,11]—and systemic illness may influence renal and haematological measurements around the procedure [10]. Nevertheless, neonatal creatinine is affected by postnatal maturation, hydration and analytical methodology; the present findings cannot establish renal injury or a causal contribution to ET receipt.

Urea and CRP differed in univariable analyses, but CRP was not included in the primary model because it is a non-specific inflammatory marker and the number of events limited model complexity; its exclusion was an analytical modelling decision, not an a priori protocol. Urea was excluded from the primary model for parsimony. ABO incompatibility and direct Coombs positivity were retained as clinically relevant haemolysis-related covariates despite non-significant adjusted estimates. Encephalopathy and IVIG were not included as baseline covariates because they can be components or consequences of the treatment pathway rather than clearly antecedent confounders. The lack of standardised reticulocyte/G6PD and haemolysis-severity data precludes detailed aetiological adjustment [2,7,21]. The absence of an independent association for ABO incompatibility is consistent with findings from an earlier Turkish ET cohort [16], whereas the Turkish Neonatal Jaundice Online Registry identified direct Coombs positivity as a risk factor in a substantially larger population [21]. These differences may reflect study size, case mix and differences in clinical management. Importantly, isoimmune haemolysis is a recognised bilirubin neurotoxicity risk factor in guideline-based management rather than a stand-alone indication for ET [2,15].

Recorded bilirubin encephalopathy was more common among infants receiving ET, but its timing relative to the procedure and standardised neurological grading could not be established. It may therefore represent a treatment indication and disease severity rather than a post-treatment event. This retrospective classification cannot establish whether ET prevented, caused or modified neurological injury. Moderate or advanced acute bilirubin encephalopathy constitutes an urgent clinical indication for ET in established treatment guidance [2,7]. Therefore, the higher recorded frequency of encephalopathy in the ET group is compatible with confounding by indication and must not be interpreted as evidence of procedural neurological harm. Earlier studies have likewise linked marked hyperbilirubinaemia with neurological morbidity [9,14,16,19,20].

After ET, total bilirubin, platelet count and total serum calcium were lower on paired measurement, whereas haemoglobin and sodium changes were not statistically significant. These observations are clinically relevant for post-exchange monitoring, but the absence of a comparable untreated control group and non-standardised sampling intervals preclude causal attribution. The measured calcium was total rather than ionised calcium; therefore, a lower total calcium value does not prove clinically significant ionised hypocalcaemia. Citrate exposure is a plausible mechanism but was not directly measured [5,6,7,11]. The marked reduction in TSB is consistent with the rapid bilirubin clearance reported in other exchange transfusion series [6,11,22]. ET removes circulating bilirubin, antibody-coated erythrocytes and antibodies while replacing circulating blood with donor components [11,12,23]. Post-exchange thrombocytopenia and decreases in calcium have been described repeatedly [5,6,7,11,24,25], and citrate-associated calcium binding offers a biologically plausible explanation [11,22]. Other cohorts have reported changes in haemoglobin after ET [6,11], emphasising that haematological effects may vary with patient characteristics and procedural practice.

The findings support careful guideline-based evaluation and monitoring rather than a new clinical prediction or treatment algorithm. The model’s out-of-fold calibration slopes (0.80 and 0.72) and exploratory nonlinearity checks reinforce the need for larger independent datasets before any predictive application. Because ET remains an invasive intervention associated with potential metabolic, haematological, infectious and haemodynamic complications [5,6,11,24,25], these data underscore the clinical value of meticulous procedural care and laboratory surveillance. Such surveillance complements, rather than replaces, timely escalation guided by bilirubin trajectories, neurological findings and recognised neurotoxicity risk factors [2,7,15].

Additional limitations include potential referral and selection bias in a tertiary unit, possible missing-data bias, non-standardised peak-TSB ascertainment and sample timing, incomplete patient-level ET procedural details, and the absence of standardised neurological grading or haemolysis-severity data. The Jaffé creatinine assay may be affected by marked bilirubin concentrations; sample-specific icteric-interference handling could not be verified. Numerous group comparisons were exploratory and unadjusted for multiplicity. The models were developed and assessed in a small cohort, with exploratory evidence of logit nonlinearity and some overfitting; external validation is absent. Total—rather than ionised—calcium was measured. Causality cannot be inferred.

The screening and exclusion counts were available for the study cohort (107 records screened; nine excluded; 98 included). Nevertheless, retrospective referral patterns and ascertainment at a single tertiary centre may limit generalisability.

## 9. Conclusions

In this tertiary neonatal cohort, peak TSB was strongly associated with receipt of ET, but its apparent discrimination partly reflects the fact that bilirubin concentration guides the treatment decision. Although creatinine showed an exploratory adjusted association, its inclusion did not demonstrate a reliable gain in discrimination, and Jaffé assay interference warrants caution. The observed reductions in bilirubin, platelet count and total calcium following ET underscore the importance of vigilant biochemical and haematological monitoring. These findings strengthen the case for timely, guideline-based assessment and careful post-exchange surveillance; they do not justify replacing established ET thresholds with cohort-derived cut-offs or deploying an unvalidated prediction model.

## Figures and Tables

**Figure 1:**
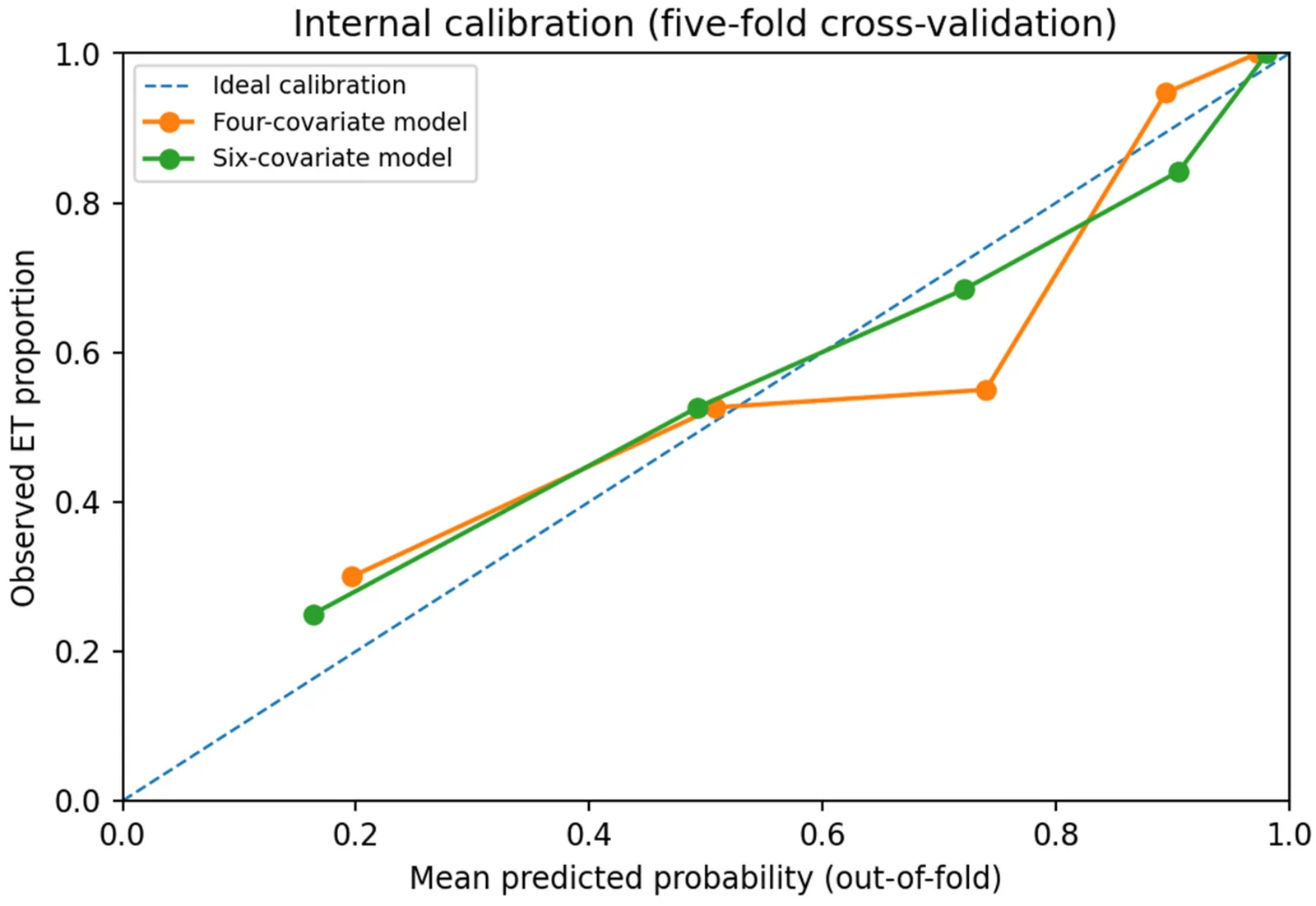
Internal calibration curves based on five-fold out-of-fold probabilities; each point represents a quintile of predicted risk. The dashed line indicates ideal calibration.

**Figure 2:**
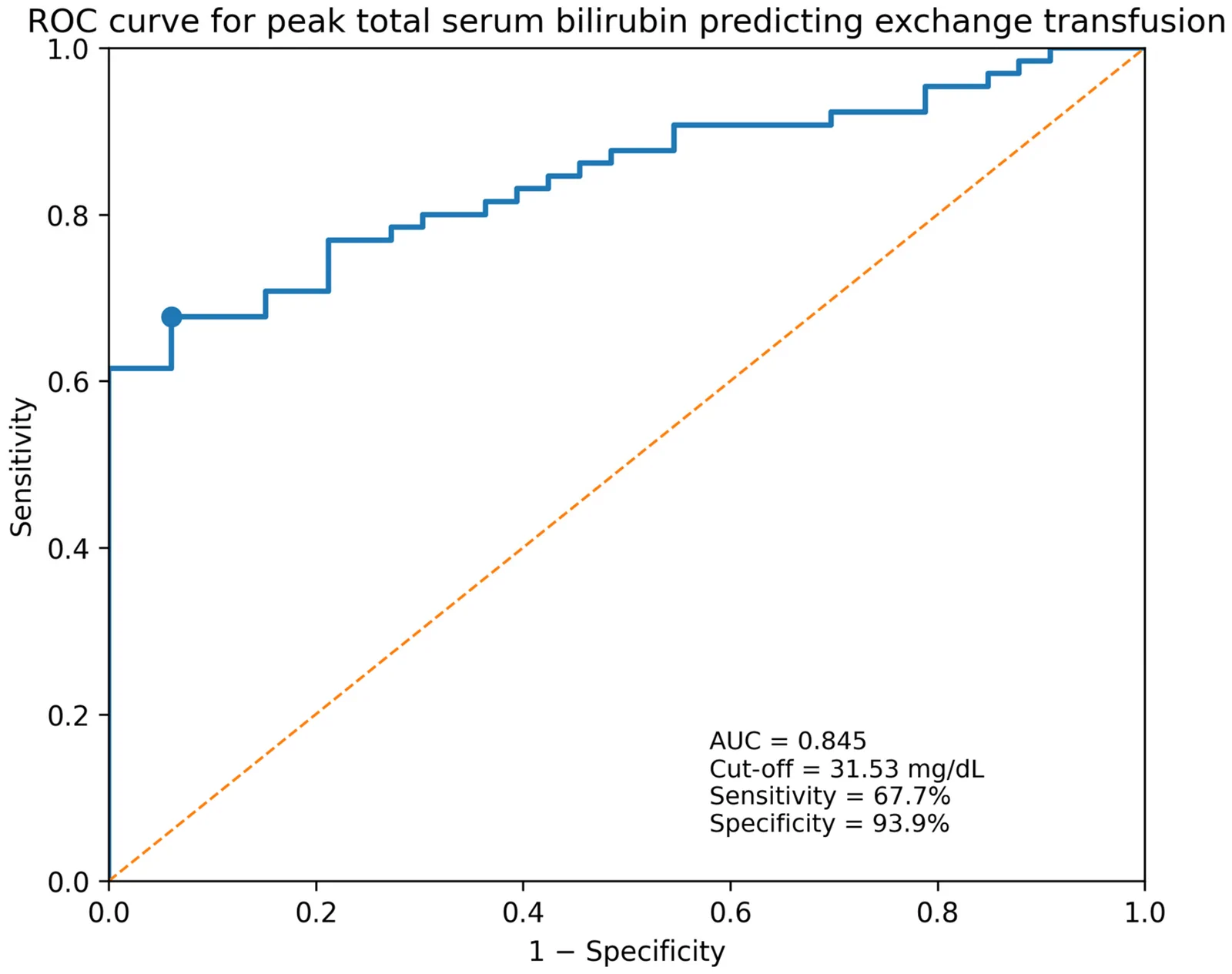
Receiver operating characteristic curve for peak total serum bilirubin alone, distinguishing infants who received exchange transfusion from those who did not (apparent AUC 0.845, 95% CI 0.770–0.919). The cohort-derived Youden threshold was ≥31.53 mg/dL. This is not a guideline treatment threshold.

**Table 1:** Baseline demographic characteristics according to exchange transfusion status.

Variable	Non-ET (*n* = 33)	ET (*n* = 65)	*p* Value
Gestational age (weeks)	38 (38–39)	38 (38–39)	0.534
Birth weight (g)	3300 (3000–3600)	3200 (2800–3500)	0.201
Postnatal age at admission (days)	4 (3–6)	5 (3–7)	0.136
Peak total bilirubin (mg/dL)	26.19 ± 3.64	33.69 ± 6.31	<0.001

Footnotes: Gestational age, birth weight and postnatal age are presented as median (interquartile range [IQR]) and were compared using the Mann–Whitney U test. Peak total bilirubin is presented as mean ± standard deviation (SD) and was compared using Welch’s independent-samples *t*-test. ET, exchange transfusion; IQR, interquartile range; SD, standard deviation.

**Table 2:** Aetiological and clinical characteristics according to exchange transfusion status.

Variable	Non-ET (*n* = 33)	ET (*n* = 65)	*p* Value
ABO incompatibility, *n* (%)	20 (60.6)	27 (41.5)	0.089
Rh incompatibility, *n* (%)	4 (12.1)	10 (15.4)	0.767
Direct Coombs positivity, *n* (%)	12 (36.4)	13 (20.0)	0.091
IVIG administration, *n* (%)	7 (21.2)	9 (13.8)	0.393
Bilirubin encephalopathy, *n* (%)	2 (6.1)	23 (35.4)	0.001
Mortality, *n* (%)	2 (6.1)	6 (9.2)	0.714

Footnotes: Data are presented as number (%). Categorical variables were compared using Pearson’s χ^2^ test or Fisher’s exact test, as appropriate. ET, exchange transfusion; IVIG, intravenous immunoglobulin.

**Table 3:** Baseline laboratory findings according to exchange transfusion status.

Variable	Non-ET (*n* = 33)	ET (*n* = 65)	*p* Value
Haemoglobin (g/dL)	15.34 ± 3.25	14.56 ± 3.41	0.273
Platelet count (×10^3^/µL)	331 (227–397)	305 (216–395)	0.475
Blood urea (mg/dL)	21.70 (14.50–33.20)	34.20 (20.55–46.90)	0.009
Serum creatinine (mg/dL)	0.54 (0.44–0.60)	0.53 (0.44–0.69)	0.309
Albumin (g/L)	36.40 (32.60–39.40)	36.80 (35.40–39.03)	0.425
Sodium (mmol/L)	140.00 (137.80–142.00)	141.00 (138.00–144.90)	0.102
Calcium (mg/dL)	9.68 ± 0.99	9.35 ± 1.02	0.119
C-reactive protein (mg/L)	0.89 (0.55–1.62)	1.40 (0.60–3.32)	0.039

Footnotes: Haemoglobin and calcium are presented as mean ± SD and were compared using Welch’s *t*-test; other laboratory variables are shown as median (IQR) and were compared using the Mann–Whitney U test. Urea and creatinine were each missing in one infant. Comparisons were exploratory; unadjusted *p* values should not be interpreted as confirmatory. ET, exchange transfusion; IQR, interquartile range; SD, standard deviation.

**Table 4:** Adjusted associations with receipt of exchange transfusion: primary and exploratory models.

Variable	Adjusted OR	95% CI	*p* Value
PRIMARY MODEL (*n* = 98)			
Peak TSB (per 1 mg/dL)	1.31	1.16–1.48	<0.001
Postnatal age (days)	0.97	0.77–1.20	0.753
ABO incompatibility	0.56	0.20–1.61	0.285
Direct Coombs positivity	0.71	0.19–2.62	0.607
EXPLORATORY SIX-COVARIATE MODEL (*n* = 97)			
Peak TSB (per 1 mg/dL)	1.37	1.18–1.59	<0.001
Postnatal age (days)	1.05	0.82–1.35	0.696
Blood urea (per 1 mg/dL)	0.99	0.95–1.03	0.695
Creatinine (per 0.1 mg/dL)	1.58	1.07–2.32	0.021
ABO incompatibility	0.58	0.19–1.77	0.341
Direct Coombs positivity	0.81	0.20–3.29	0.770

Footnotes: Adjusted ORs (95% CI) were estimated using logistic regression. The primary model (*n* = 98) excluded urea and creatinine; the six-covariate model (*n* = 97) is exploratory. Creatinine is expressed per 0.1 mg/dL for clinical readability. Primary-model VIF range: 1.03–1.24; six-covariate range: 1.03–1.98. Neither model is externally validated. CI, confidence interval; ET, exchange transfusion; OR, odds ratio; TSB, total serum bilirubin.

**Table 5:** Paired comparison of laboratory parameters before and after exchange transfusion.

Variable	Before ET	After ET	Paired Change (95% CI)	*p* Value
Haemoglobin (g/dL)	14.56	14.05	−0.51 (−1.34 to 0.32)	0.234
Platelet count (×10^3^/µL)	318.0	119.7	−198.30 (−236.41 to −160.18)	<0.001
Serum sodium (mmol/L)	141.0	140.9	−1.0	0.187
Serum calcium (mg/dL)	9.40	8.49	−0.82	0.002
Total bilirubin (mg/dL)	33.69	14.69	−18.99 (−20.39 to −17.59)	<0.001

Footnotes: Haemoglobin, platelet count and total bilirubin are presented as arithmetic means; paired differences were assessed using the paired Student’s *t*-test and are reported with 95% confidence intervals. Sodium and calcium are presented as medians; paired differences were assessed using the Wilcoxon signed-rank test and are reported as median paired changes. Complete paired data were available for 65 infants, except for platelet count (*n* = 64). CI, confidence interval; ET, exchange transfusion.

## Data Availability

The data presented in this study are available from the corresponding author upon reasonable request. The data are not publicly available because they contain clinical information derived from patient medical records.

## Abbreviations

aOR, adjusted odds ratio; AUC, area under the curve; CI, confidence interval; CRP, C-reactive protein; ET, exchange transfusion; IVIG, intravenous immunoglobulin; IQR, interquartile range; ROC, receiver operating characteristic; SD, standard deviation; TSB, total serum bilirubin; VIF, variance inflation factor.
